# Supplementary material for: CRISPR/Cas9-Mediated Editing in FAD2 Gene to Enhance Oil Quality in Soybean [Glycine max (L.) Merrill]
Source: PLoS One. 2026 Feb 13;21(2):e0342660. doi: 10.1371/journal.pone.0342660 (PMC12904588; doi:10.1371/journal.pone.0342660)
Supplement: S1 File — (PDF) [file pone.0342660.s001.pdf]

Supplementary Information

CRISPR/Cas9-Mediated Editing of the FAD2 Gene to Enhance Oil Quality in Soybean (Glycine max L.)

Supplementary Information

Author:

Dr. Sunil Hajare, PhD

Dilla University, Dilla, Ethiopia

S1_File.pdf → 13 figures (S1, S1G, S2-S13)

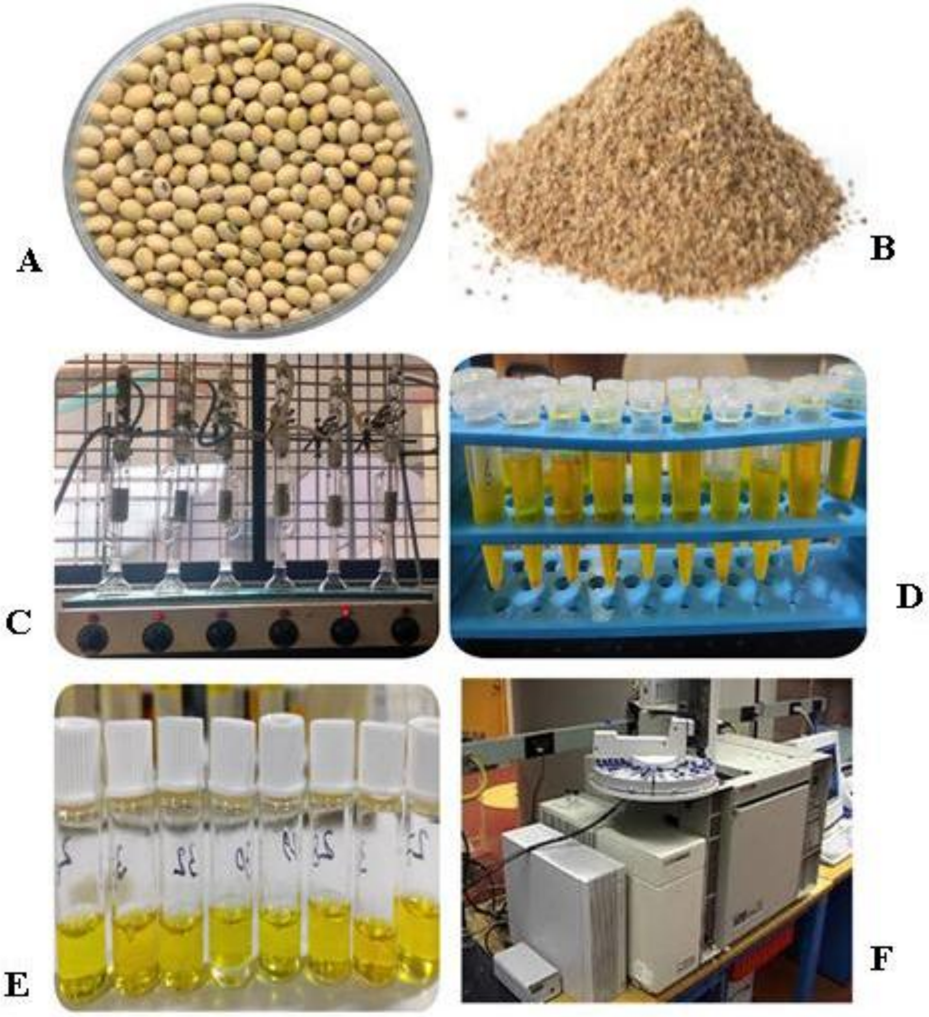

Figure S1 Determination of Total Oil Content by Soxhlet method and Preparation of FAME samples from the soybean matrices

A) Soyabean seeds B) Soyabean seed powder C) Soxlet appratus D) Oil extracted by Soxlet method E) Derivatization of oil for GCMS F) GC-MS used for fatty acid Profiling

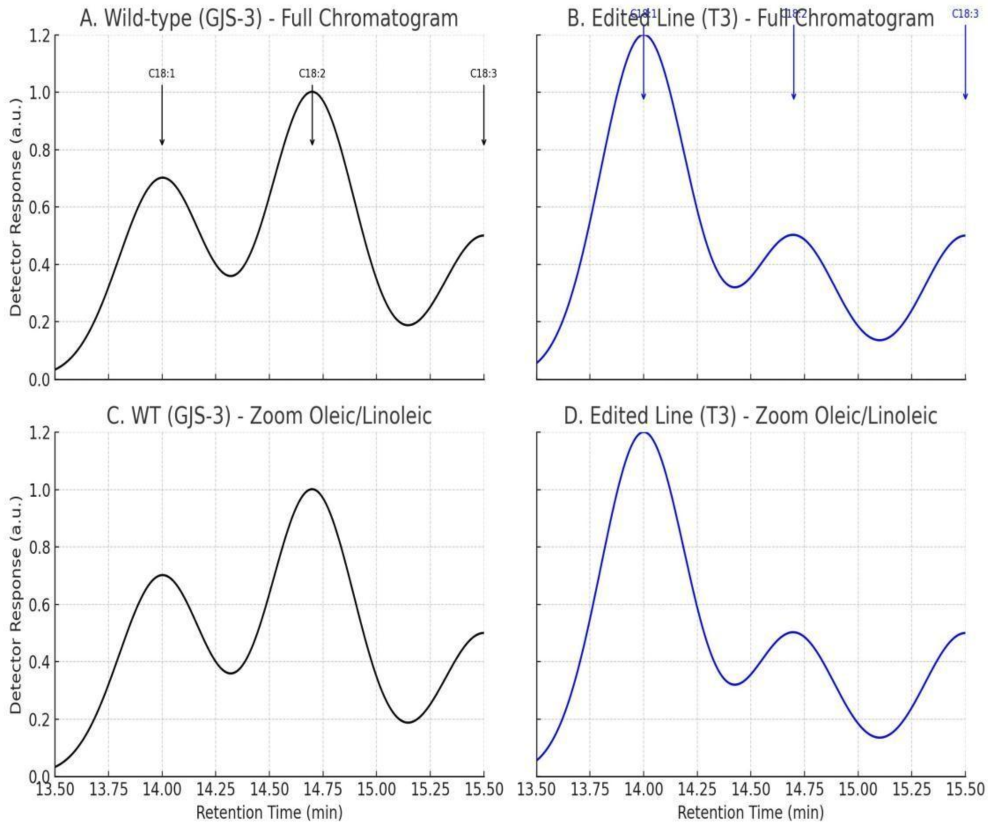

Figure S1 **G:** Representative GC–MS chromatograms of fatty acid methyl esters (FAMEs) from soybean oil.

(A) Wild-type GJS-3 (full chromatogram), (B) edited line T3 (full chromatogram), (C) enlarged oleic (C18:1) and linoleic (C18:2) region in wild-type, and (D) the corresponding region in edited line T3. Peaks are annotated as C16:0 (palmitic), C18:0 (stearic), C18:1 (oleic), C18:2 (linoleic), C18:3 (linolenic), and C17:0 (internal standard). The internal standard (C17:0) was used for quantification, and calibration curves from certified FAME standards were applied to determine fatty acid composition.

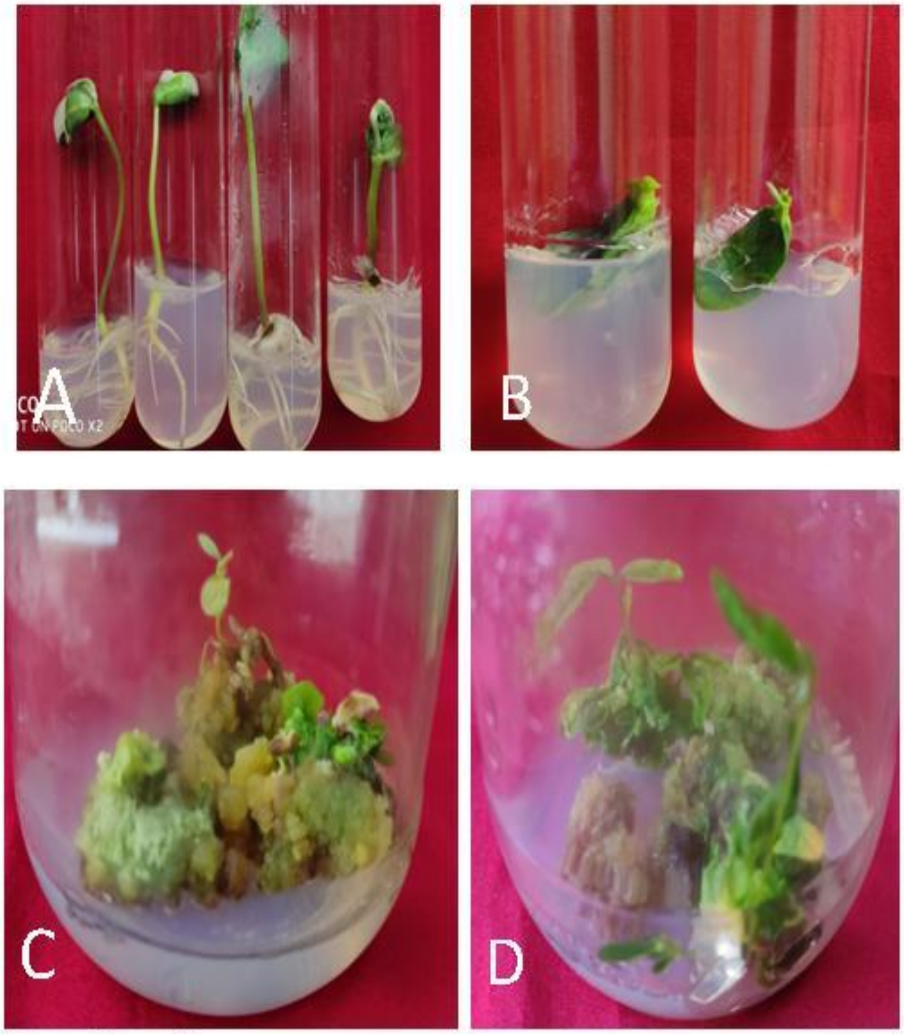

Supplementary Figure S2

Effect of 2,4,D and BAP on Callus induction in Soybean cv. GJS-3:

A) 7 days old invitro growth of soyabean plants (MS + 0.0mg/l 2, 4 D + 0.0 mg/l BAP)

B) Inoculated Soybean cotyledonory explants on shoot regeneration media (MS + 3.0mg/l 2, 4 D + 3.0 mg/l BAP) at 10 day in culture

C) Callus induction from cotyledonory node explants after 21 days on culture media (MS + 3.0mg/l 2, 4, D + 3.0 mg/l BAP)

D) Shoot initiation from cotyledon explants after 32 days of culturing on (MS + 3.0mg/l 2, 4, D + 3.0 mg/l BAP)

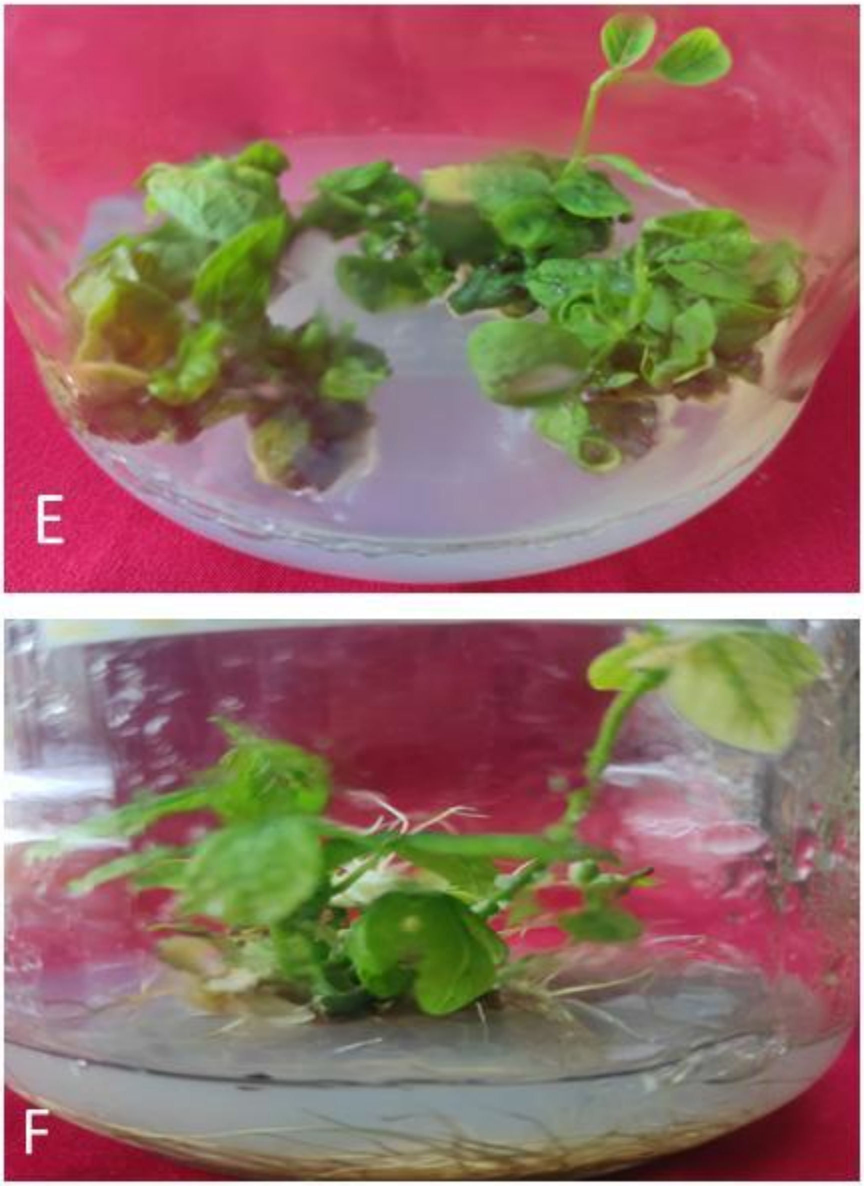

Supplementary Figure S3

E -F) Shoot elongation and multiplication after 45 days on culture media + 3.0mg/l 2, 4, D + 3.0 mg/l BAP)

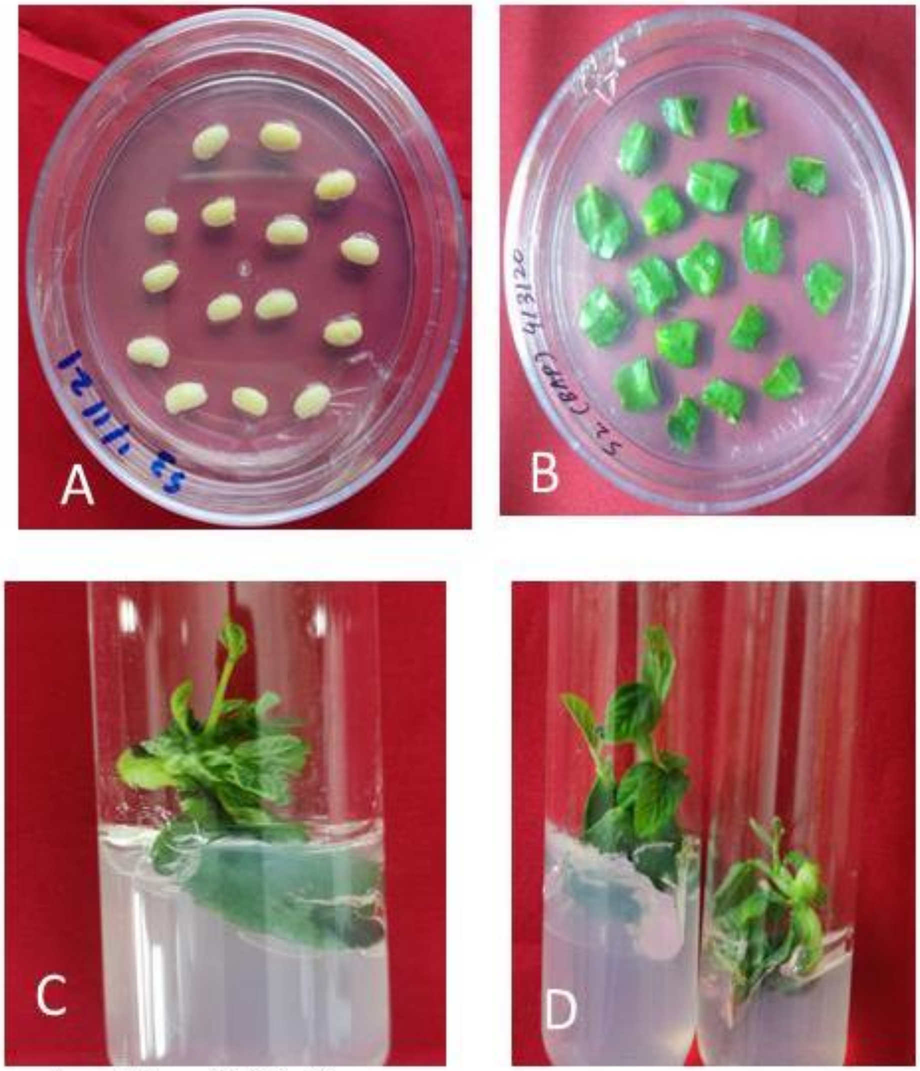

Supplementary Figure S4 (A-G)

Effect of BAP on shoot regeneration from cotyledon explants in Soybean cv. GJS-3:

A) Germinated soybean seeds after 6 to 8 days in an MS medium without hormones.

B) Cotyledonory node explants inoculated on shoot regeneration media (MS + 3.0 mg/l BAP) on day 10 of culturing

C-D) Shoot initiation from cotyledon ory node explants after 25 days of culturing on (MS + 3.0mg/l BAP)

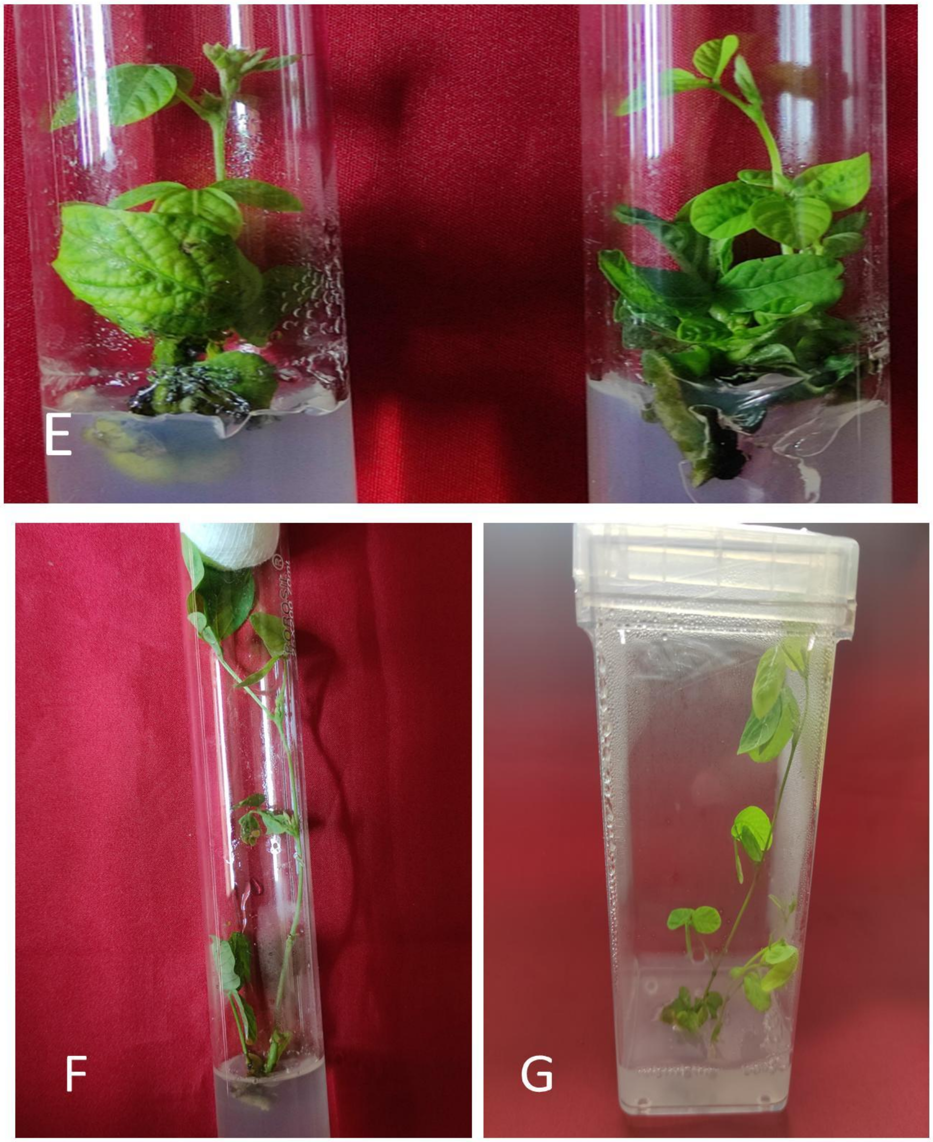

E -F-G) Shoot multiplication and elongation after 45 days on culture media (MS + 3.0 mg/l BAP)

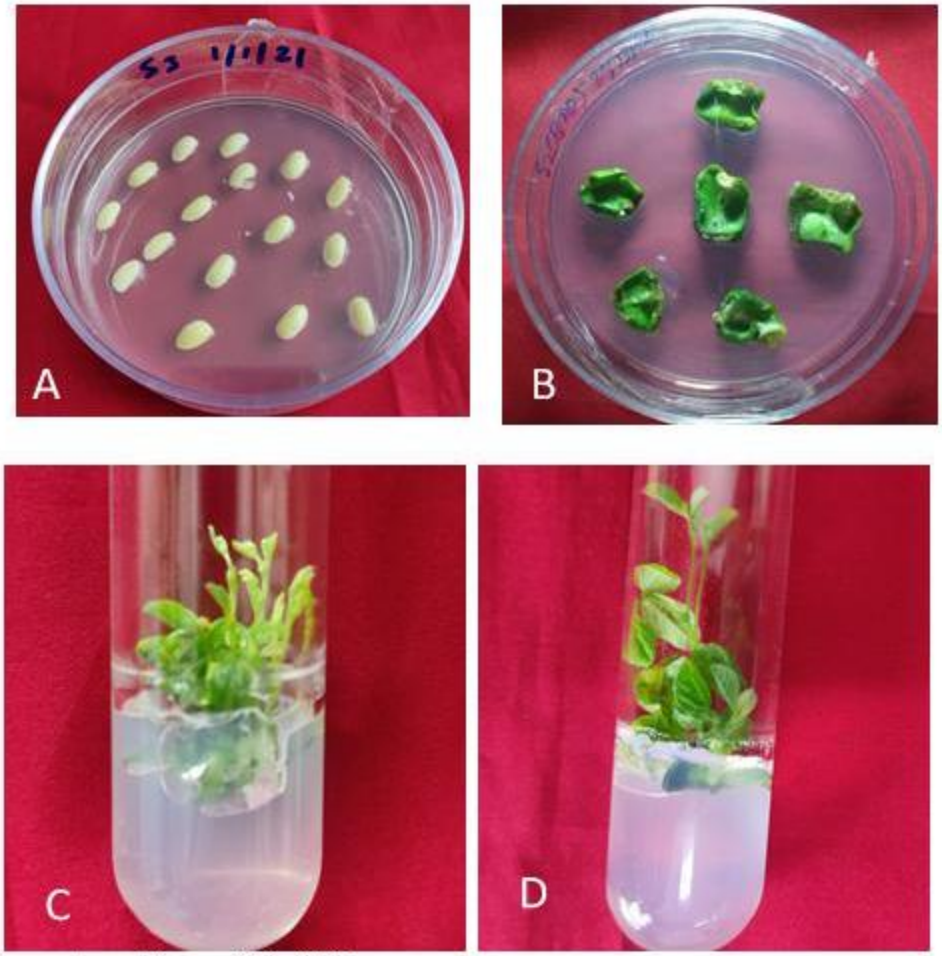

Supplementary Figure S5, A-H

Effect of BAP and GA on multiple shoot initiation and elongation

A) Germinated soybean seeds after 6 to 8 days in an MS medium without hormones.

B) Inoculated Soybean cotyledonory explants on shoot regeneration media (MS + 3.0 mg/l BAP) at 14 day in culture

C-D) Multiple shoot initiation from cotyledonory node explant after 35 days on culture media (MS + 3.0mg/l BAP + 1.0 mg/l GA)

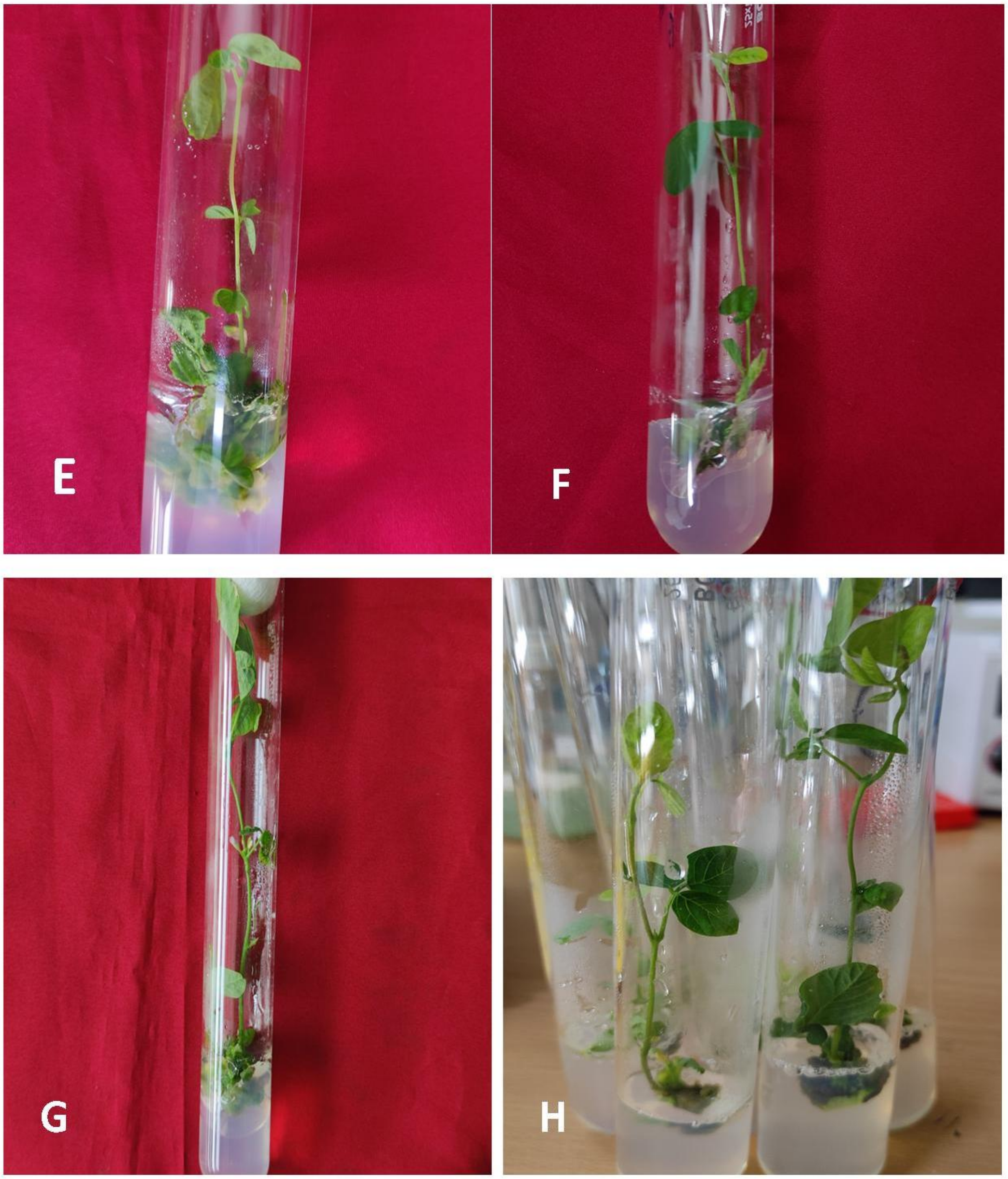

E-F-G-H) Shoot elongation from cotyledonory node explant after 45 days on culture media (MS + 3.0mg/l BAP + 1.0 mg/l GA)

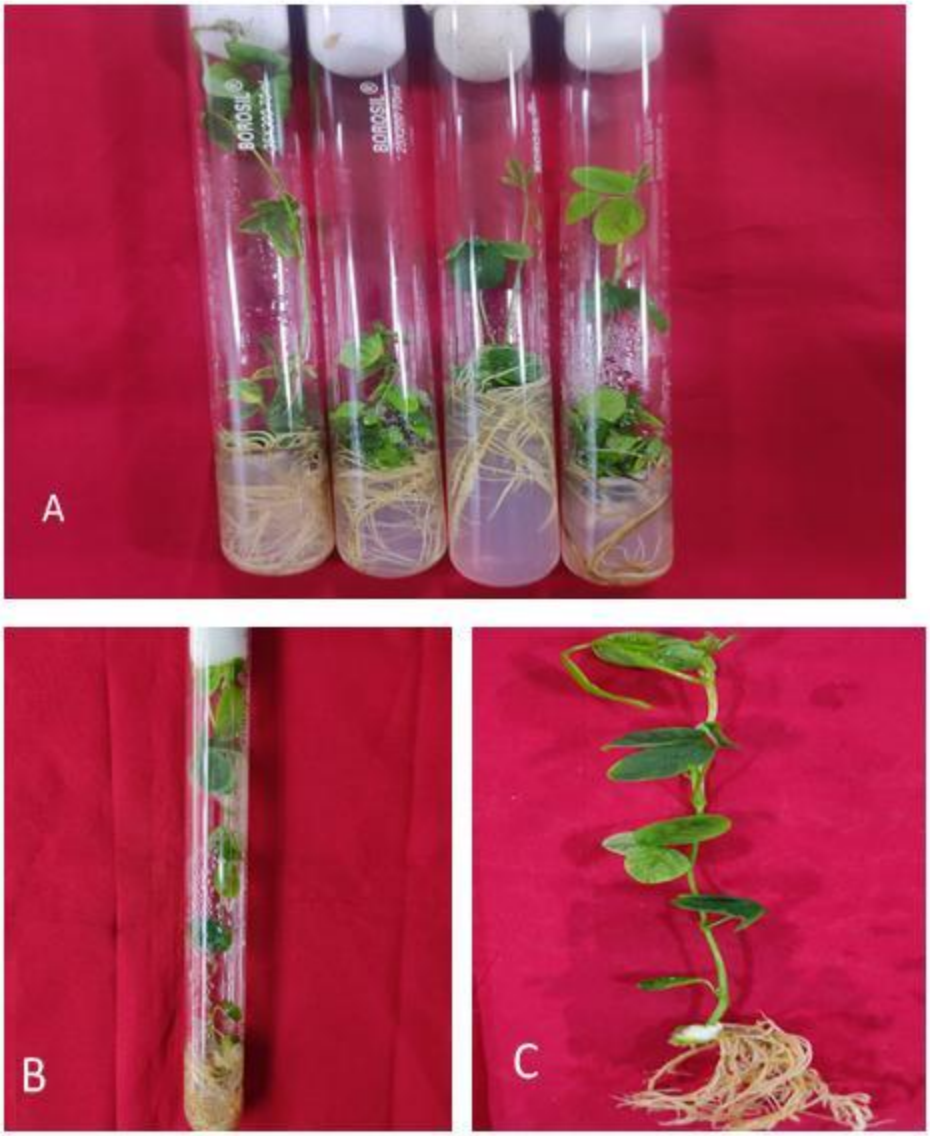

Supplementary Figure S6

Effect of IBA on root regeneration from multiple shoot explants in Soybean cv. GJS-3

A) Multiple root initiation in *in vitro* developed shoots
B) Fully grown plantlets showing profuse rooting
C) Regenerated plant ready for acclimatisation

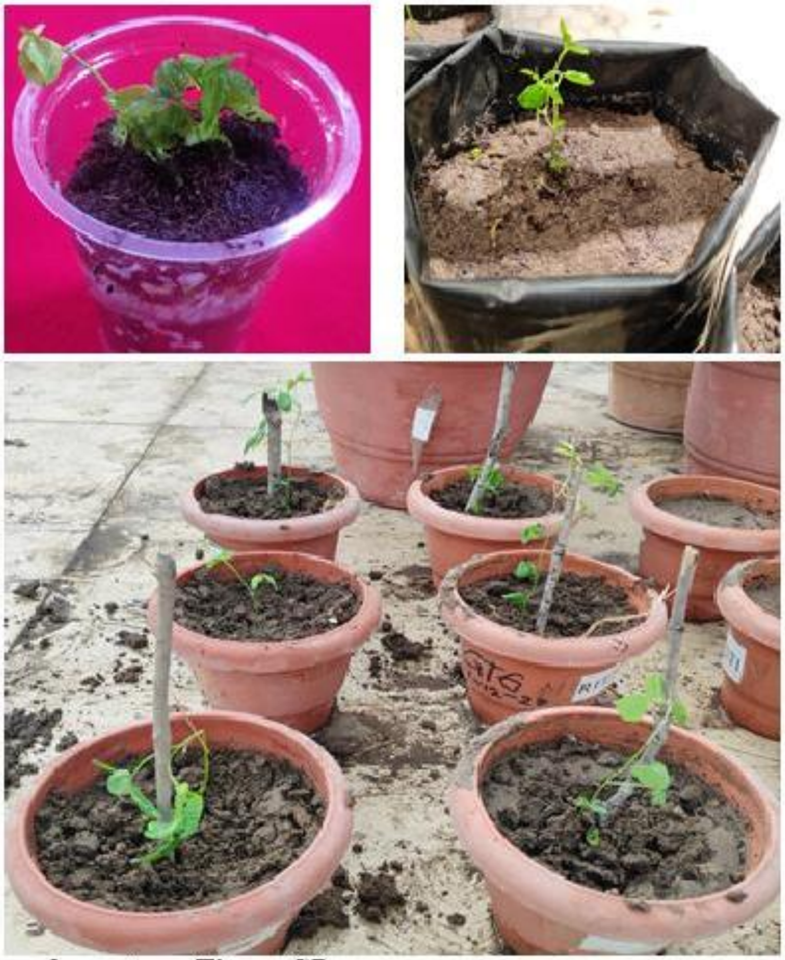

Supplementary Figure S7

Hardening of regenerated plantlets of soybean [*Glycine max* (L.) Merrill]" cv. GJS -3

TAACCATATACTAATATTTGCTTGTATTGATAGCCCCTCCGTTCC
CAAGAGTATAAAACTGCATCGAATAATACAAGCCACTAGGCAT
GGTAAATTAAATTGTGCCTGCACCTCGGGATATTTCATGTGGGG
TTCATCATATTTGTTGAGGAAAAGAAACTCCCGAAATTGAATT
ATGCATTTATATATCCTTTTTCATTTCTAGATTTCCTGAAGGCTT
AGGTGTAGGCACCTAGCTAGTAGCTACAATATCAGCACTTCTC
TCTATTGATAAACAATTGGCTGTAATGCCGCAGTAGAGGACGA
TCACAACATTTCGTGCTGGTTACTTTTTGTTTTATGGTCATGATT
TCACTCTCTCTAATCTCTCCATTCATTTTGTAGTTGTCATTATCT
TTAGATTTTTCACTACCTGGTTTAAAATTGAGGGATTGTAGTTC
TGTTGGTACATATTACACATTCAGCAAAACAACTGAAACTCAA
CTGAACTTGTTTATACTTTGACACAGGGTCTAGCAAAGGAAAC
AACAATGGGAGGTAGAGGTCGTGTGGCCAAAGTGGAAGTTCA
AGGGAAGAAGCCTCTCTCAAGGGTTCCAAACACAAAGCCACC
ATTCACTGTTGGCCAACTCAAGAAAGCAATTCCACCACACTGC
TTTCAGCGCTCCCTCCTCACTTCATTCTCCTATGTTGTTTATGAC
CTTTCATTTGCCTTCATTTTCTACATTGCCACCACCTACTTCCAC
CTCCTTCCTCAACCCTTTTCCCTCATTGCATGGCCAATCTATTG
GGTTCTCCAAGGTTGCCTTCTCACTGGTGTGTGGGTGATTGCT
CACGAGTGTGGTCACCATGCCTTCAGCAAGTACCAATGGGTTG
ATGATGTTGTGGGTTTGACCCTTCACTCAACACTTTTAGTCCCT
TATTTCTCATGGAAAATAAGCCATCGCCGCCATCACTCCAACA
CAGGTTCCCTTGACCGTGATGAAGTGTTTGTCCCAAAACCAA
AATCCAAAGTTGCATGGTTTTCCAAGTACTTAAACAACCCTCT
AGGAAGGGCTGTTTCTCTTCTCGTCACACTCACAATAGGGTGG
CCTATGTATTTAGCCTTCAATGTCTCTGGTAGACCCTATGATAGT
TTTGCAAGCCACTACCACCCTTATGCTCCCATATATTCTAACCG
TGAGAGGCTTCTGATCTATGTCTCTGATGTTGCTTTGTTTTCTG
TGACTTACTCTCTCTACCGTGTTGCAACCCTGAAAGGGT

Supplementary Figure, S8 : Sequence of the FAD2 gene (973 bp) showing the designed gRNA (green) with adjacent PAM (red). Primers were designed using NCBI Primer-BLAST.

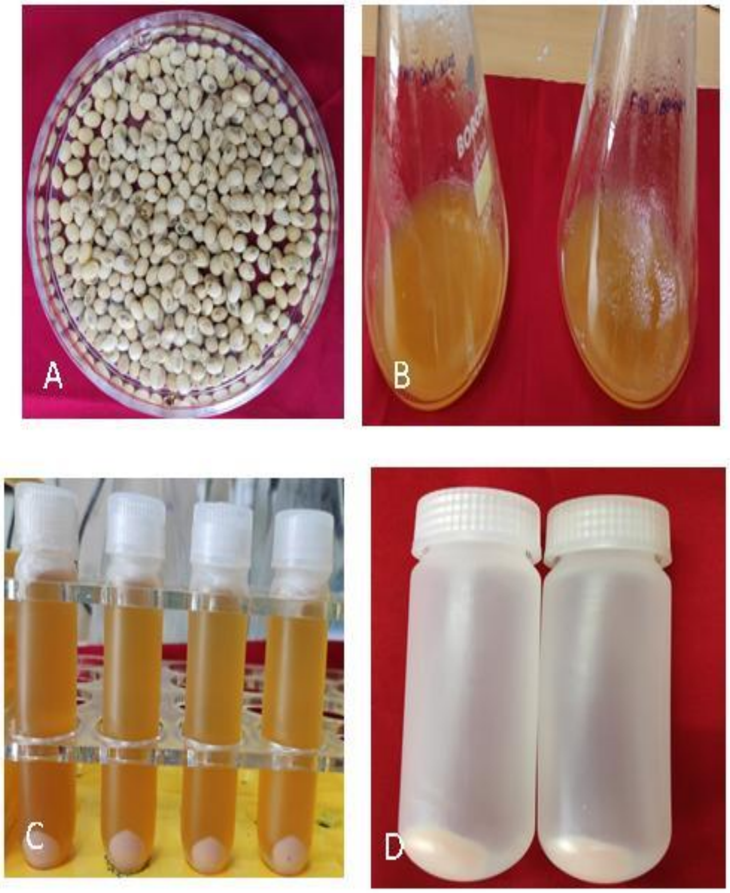

Supplementary figure S9

The experimental process of Agrobacterium-mediated transformation using half-seed soybean explants

A) Seed imbibition
B) Preparation of Agrobacterium
C) Agrobacterium at the bottom of okrich tube after centrifugation
D) Agrobacterium after supernatant removed

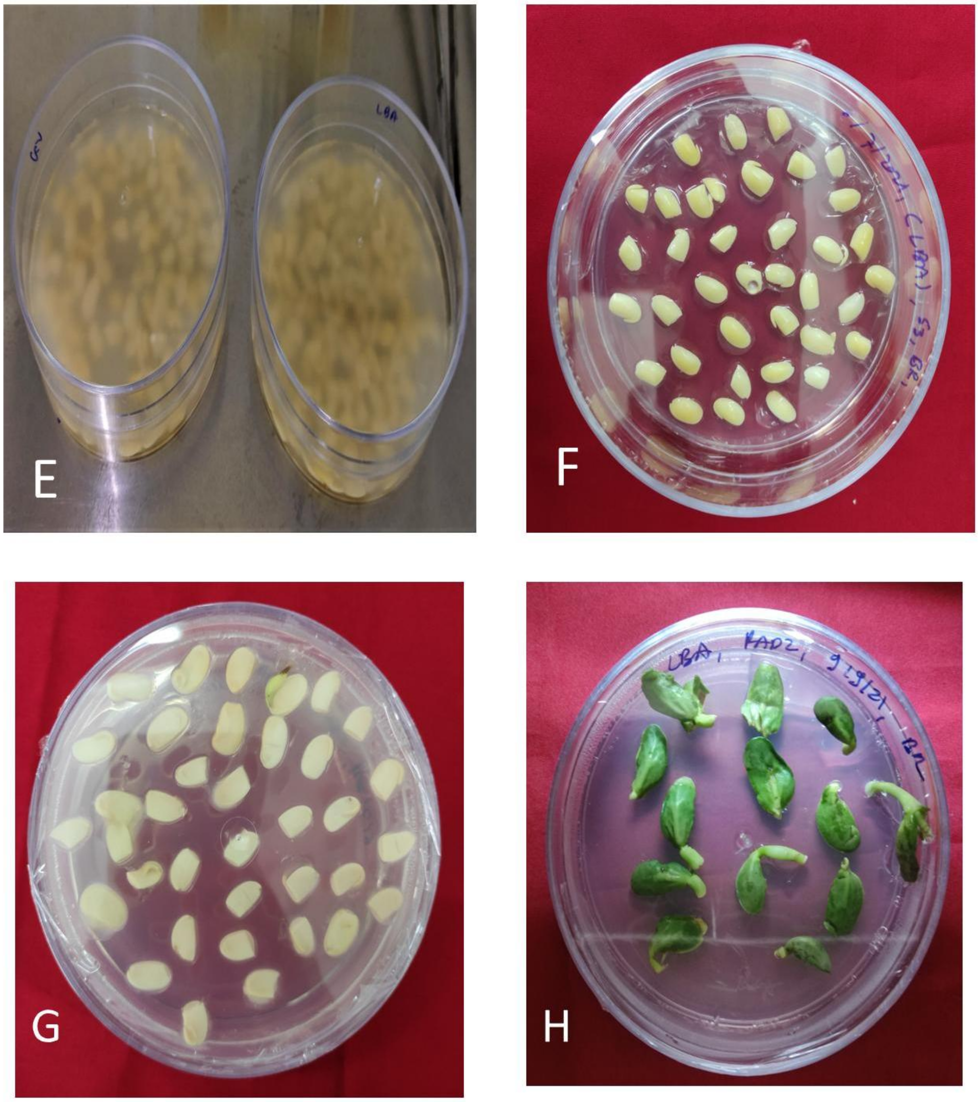

E) Cotyledonory node incubation with agrobacterium
F)Agrobacterium infection of half-seed cotyledonary explants.
G) Co-cultivation.
H) Cotyledonory node shoot induction

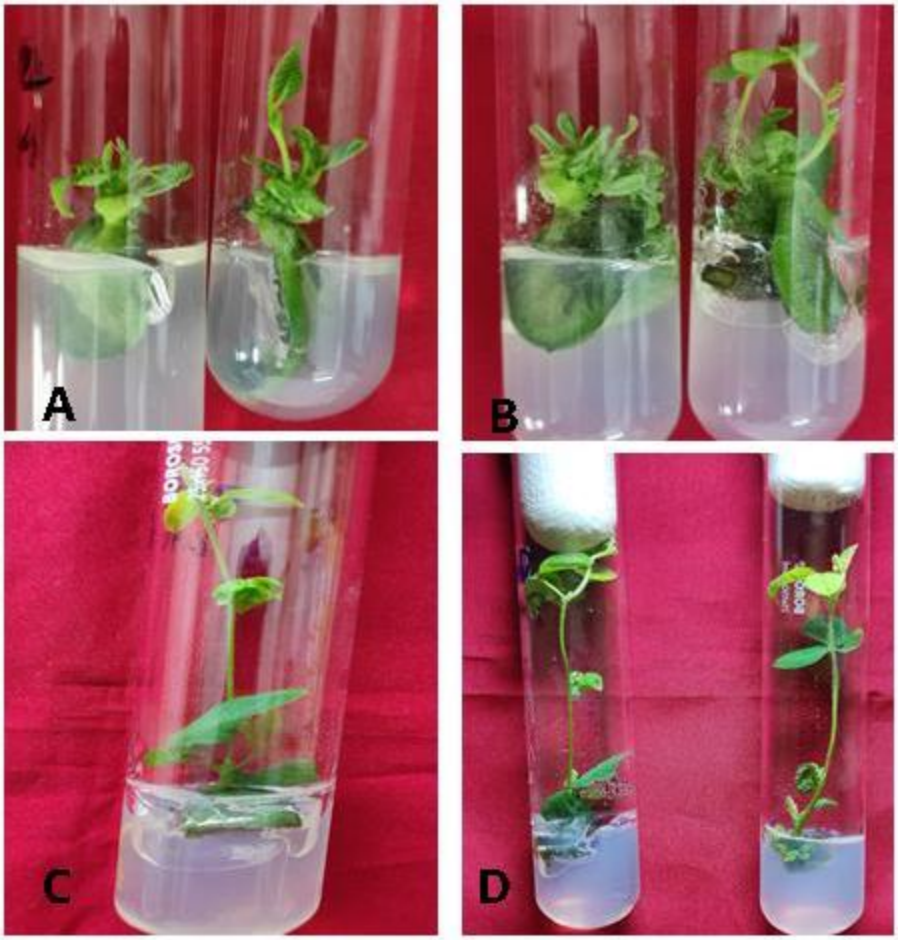

Supplementary Figure S10

A-B) Shoot proliferation from cotyledon ex-plants after 4th week of culturing

C-D) Elongation of *in vitro* regenerated shoots

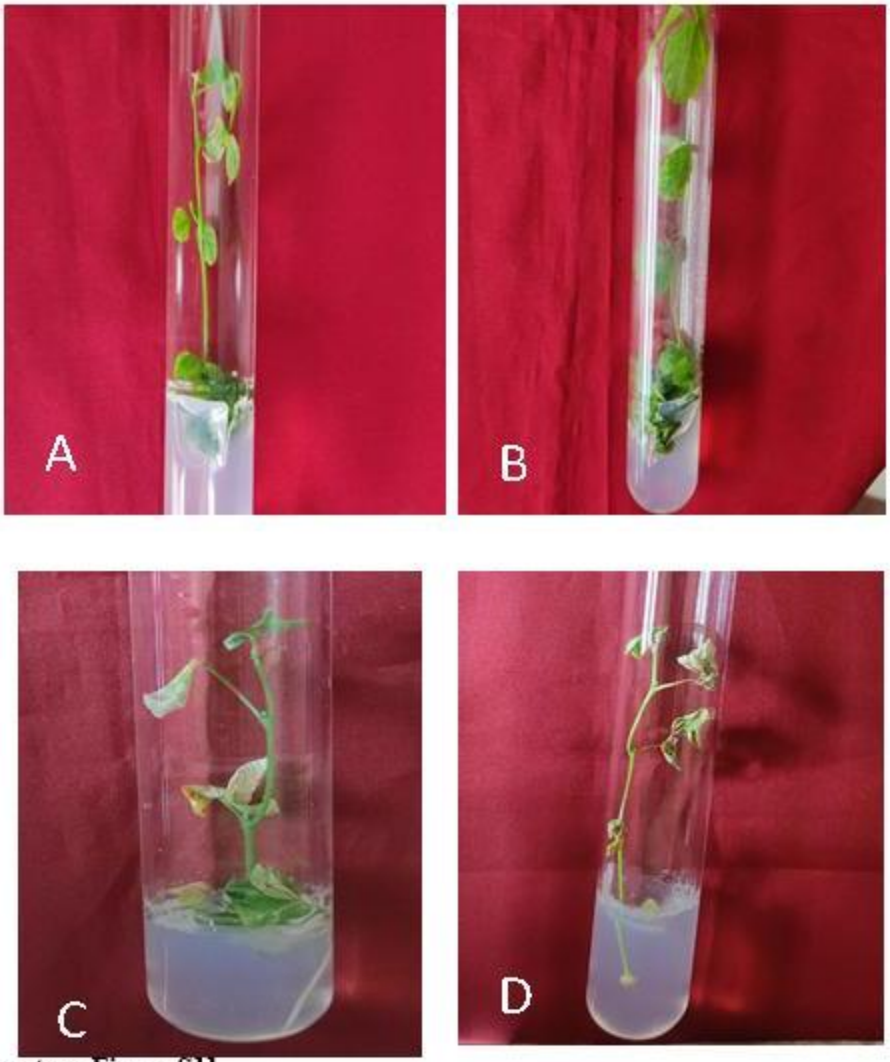

Supplementary Figure S11

Hygromycin sleection of transformed shoots

A) *In vitro* regenerated shoots kept on Hygromycin selection media

B) Hygromycin resistant transformed shoot after 4 days

C-D) Hygromycin susceptible shoot on selection media after 4 days

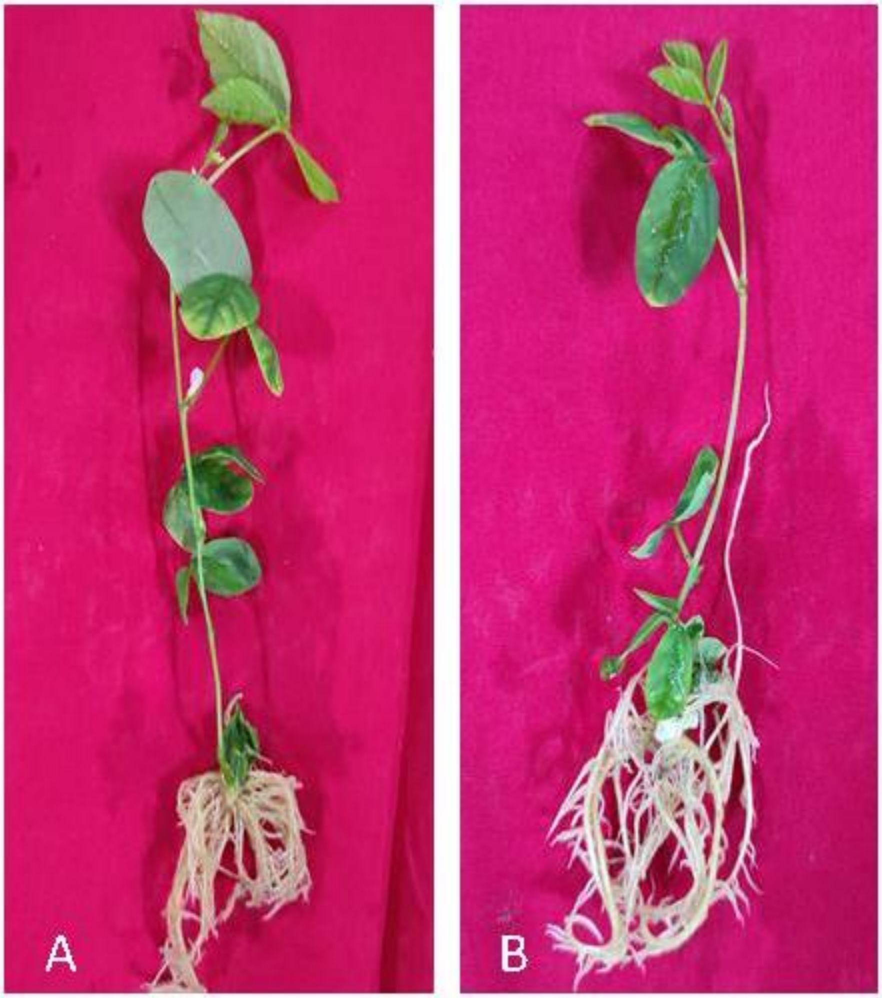

Supplementary Figure S12, A-B

. A-B) *In vitro* regenerated Soybean plants with well developed root system

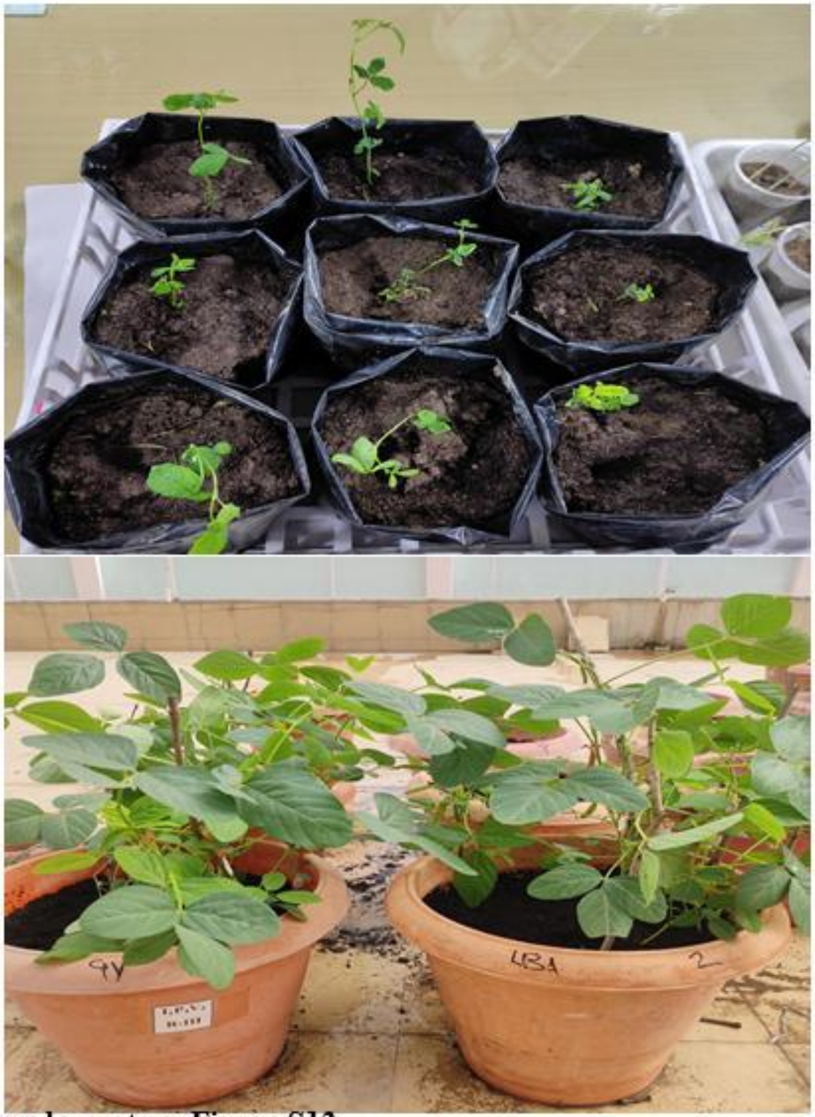

Supplementary Figure S13
Hardening and acclimatization of *in vitro* regenerated transformed plants

C) Hardened soybean shoots in potting mixture
