# Supplementary material for: CRISPR/Cas9-Mediated Editing in FAD2 Gene to Enhance Oil Quality in Soybean [Glycine max (L.) Merrill]
Source: PLoS One. 2026 Feb 13;21(2):e0342660. doi: 10.1371/journal.pone.0342660 (PMC12904588; doi:10.1371/journal.pone.0342660)
Supplement: S2 File — (PDF) [file pone.0342660.s002.pdf]

Supplementary Information

CRISPR/Cas9-Mediated Editing of the FAD2 Gene to Enhance Oil Quality in Soybean (Glycine max L.)

Supplementary Information

Author:

Dr. Sunil Hajare, PhD

Dilla University, Dilla, Ethiopia

S2_File.pdf → 11 tables (S1-S11)

Table S1: List of Genotype selected for fatty acid profiling

Sr. No	Genotype name	Remarks	Sr. No	Genotype name	Remarks
1	G.Soy 1	Released variety	21	AGS 93	Black seeded
2	G.Soy 2	Released variety	22	PBN 107	Black seeded
3	GJS 3	Released variety	23	DS 64-6	Green seeded
4	KB 74	Black seeded	24	AGS 84	Green seeded
5	AS 3	LSVT entry	25	PK 781	Brown seeded
6	AS 15	LSVT entry	26	JB 5-2	Black seeded
7	PS 1634	Yellow seeded	27	PK 942	Green seeded
8	AMRS 258	LSVT entry	28	DS 178	Black seeded
9	JS 335	Released variety	29	KS 166	Brown seeded
10	AS 16	LSVT entry	30	AS 151	LSVT entry
11	DS 83-12	Black seeded	31	AS 14	SSVT entry
12	J 606	Black seeded	32	AUKS 176	Yellow seeded
13	AGS 112	Green seeded	33	J 15-20-2	Black seeded
14	JS 81-1619	Yellow seeded	34	JS 20-29	Released variety
15	DS 84-3	Green seeded	35	EC 93318	Green seeded
16	JS 72-128	Brown seeded	36	BR 7B	Brown seeded
17	J 556	Brown seeded	37	J 245	Brown seeded
18	JD(SH) 131	Black seeded	38	NRC 138	Yellow seeded
19	J 222	Black seeded	39	J 339	Black seeded
20	EC 93741	Yellow seeded	40	EC 1933	Brown seeded

Note on experimental design: For regeneration experiments using these genotypes, biological replicates represent independent culture batches from different parent plants initiated at different times. Statistical unit = batch mean (n=3); explants per batch (n=10) provide within-batch precision estimates but are not treated as independent replicates to avoid pseudoreplication

Table S2: Primer sequence of FAD2 gene

Sl. NO	Name of the primer	Primer Sequence 5' -3'	Tm (ºC)
1	Fw-FAD2-Soyabean	AGCAATTCCACCACACTGCT	59
2	Rv-FAD2-Soyabean	GAGGCACTCTCTCGCTTCTC	59

Table S3: gRNA sequence information

Target Sequence:5'-3'	PAM	Specificity	Chromosome
TTCTCGTCACACTCACAATAGG	AGG	97.6	10

Table S4: List of plasmid isolation alkaline stock solutions

	**Stock concentration**	**Working concentration**
Solution I	Glucose (0.5M)<br>Tris HCl (1M) (PH 8.0) EDTA<br>(0.5M) (PH 8.0)	25 Mm<br>10 mM<br>50 mM
Solution II	10 % SDS<br>1N NaOH	1%<br>0.2 N
Solution III	Glacial acetic acid<br>5M potassium acetate	11.5 %<br>3 mM potassium acetate

Table S5: TAE buffer stock solution preparation

**50X TAE buffer (1 liter) stock solution**	
Tris base	242.0 g
EDTA 0.5M (PH 8.0)	100 ml
Glacial acetic acid	57.1 ml
Working concentration 1X	

Table S6: Reaction set up for restriction enzyme digestion of binary vector pRGEB31

**Reagent**	**Volume**
pRGEB31 Plasmid	3 µl
BsaI Enzyme	1 µl
Cut smart buffer (10X)	2 µl
H20	15 µl
Total volume	20 µl

Table S7: Reaction setup for polynucleotide kinase reaction for dsDNA formation

**Reagent**	**Volume**
FAD2 ssDNA_F 100 µM	1 µl
FAD2 ssDNA_R 100 µM	1 µl
10x T4 ligase buffer	1 µl
T4 PNK enzyme	1 µl
H2O	5 µl
Total	10 µl

Table S8: PCR conditions for polynucleotide kinase reaction for dsDNA formation

37 ºC	60 min
95 ºC	10 min
Cool down to 25 ºC at 0.1 ºC/sec	

Table S9: Reaction set up for ligation of FAD2 sgRNA into pRGEB31 vector

**Reagent**	**Volume**
FAD2 oligo duplex	2 µl
BsaI digested Plasmid pRGEB31vector	2.5 µl
10x T4 ligase buffer	1 µl
T4 ligase enzyme	1 µl
H2O	3.5 µl
Total volume	10 µ

Table S10 — Farmer gross benefit (USD/ha)

Oil_kg_per_ha	$0.05/lb	$0.10/lb	$0.15/lb	$0.20/lb	$0.25/lb
100	10.00	20.00	30.00	40.00	50.00
200	20.00	40.00	60.00	80.00	100.00
300	30.00	60.00	90.00	120.00	150.00
400	40.00	80.00	120.00	160.00	200.00
500	50.00	100.00	150.00	200.00	250.00

Table S11- Processing savings (component ranges and totals, USD per tonne)

- Hydrogen: 3.6 / 6.3 / 9.0 (Low/Mid/High)
- Catalyst: 1.0 / 5.5 / 10.0
- Energy & Utilities: 1.0 / 5.5 / 10.0
- Labour & Maintenance: 5.0 / 17.5 / 30.0
- Amortized Capital/Overhead: 5.0 / 12.5 / 20.0
- Total processing_savings (USD/t): Low = 15.6; Mid = 47.3; High = 79.0

Processing savings converted to USD/ha (for oil yields 100–500 kg/ha)

Oil_kg_per_ha	Savings Low ($/ha)	Savings Mid ($/ha)	Savings High ($/ha)
100	1.56	4.73	7.90
200	3.12	9.46	15.80
300	4.68	14.19	23.70
400	6.24	18.92	31.60
500	7.80	23.65	39.50

Sources: Component cost ranges derived from UNIDO Industrial Development Report 2020 (Chapter 4, pp. 89-112), Bailey's Industrial Oil and Fat Products 6th ed. (2005, Vol. 5, Ch. 5, pp. 213-245), and United Soybean Board industry data (2022). Low/mid/high scenarios reflect variation in facility scale, technology vintage, and regional conditions. Indian facilities may experience lower labor costs and variable energy pricing. Estimates are illustrative; facility-specific analysis recommended.
