# Supplementary figures and images for: CRISPR/Cas9-Mediated Editing in FAD2 Gene to Enhance Oil Quality in Soybean [Glycine max (L.) Merrill]
Source: PLoS One. 2026 Feb 13;21(2):e0342660. doi: 10.1371/journal.pone.0342660 (PMC12904588; doi:10.1371/journal.pone.0342660)

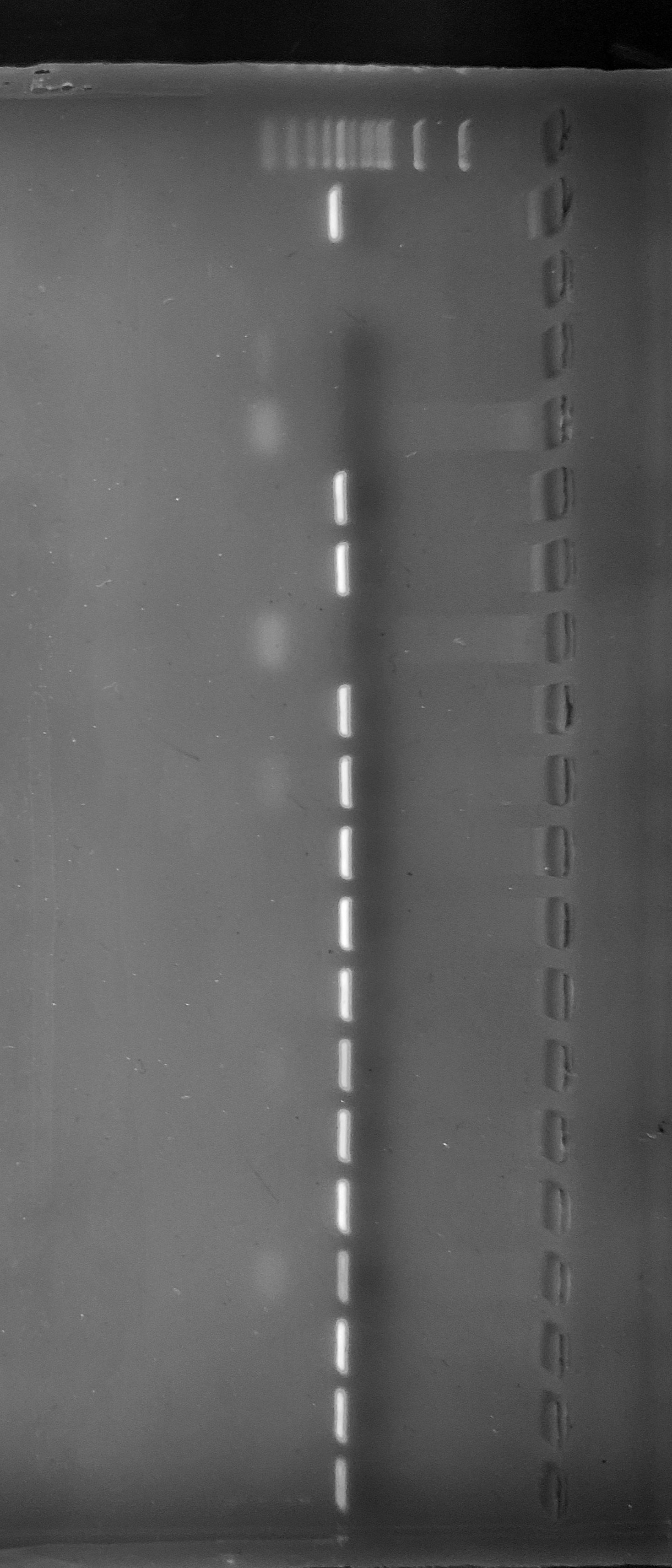

Supplement: S3 File — (JPG) [file pone.0342660.s003.jpg]

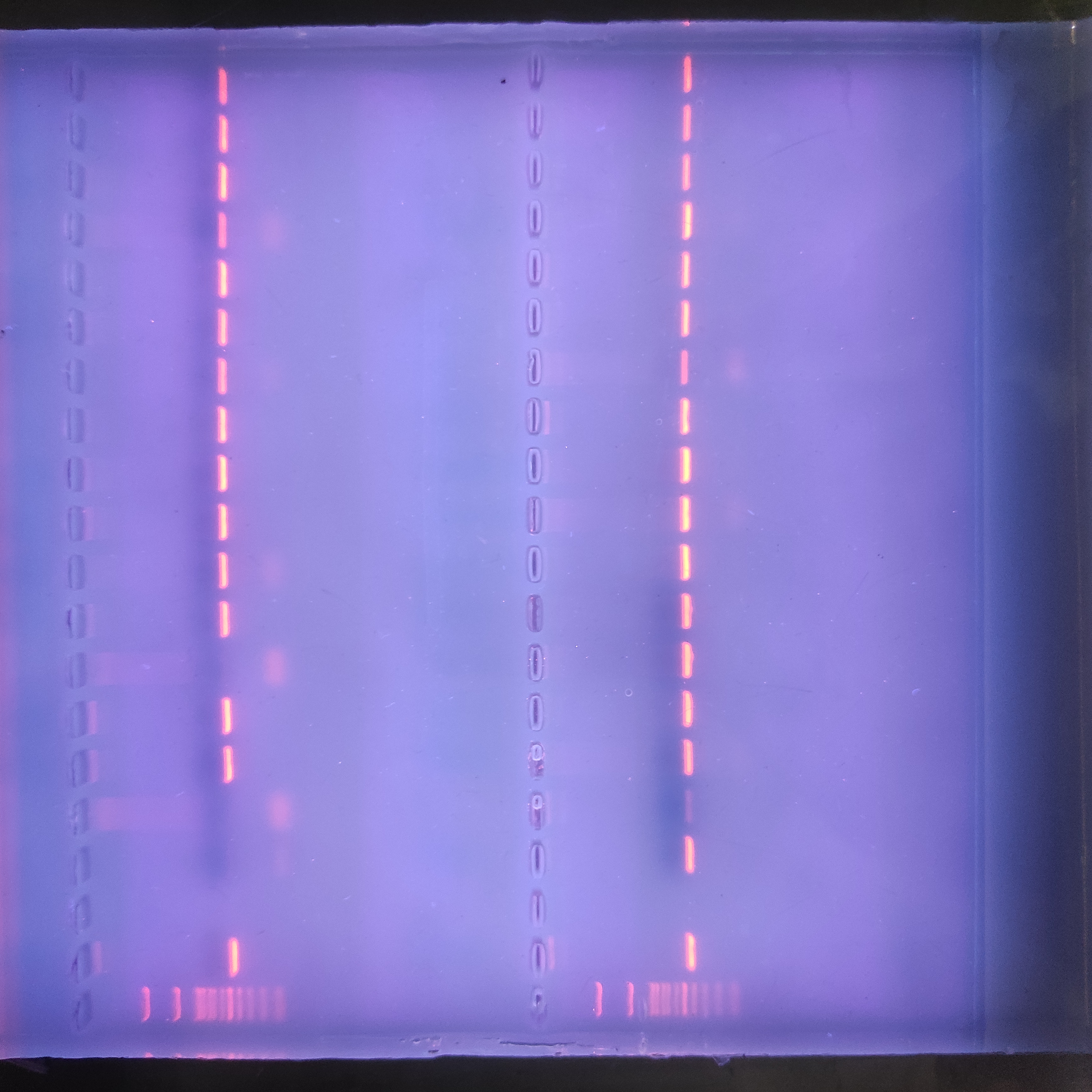

Supplement: S4 File — (JPG) [file pone.0342660.s004.jpg]

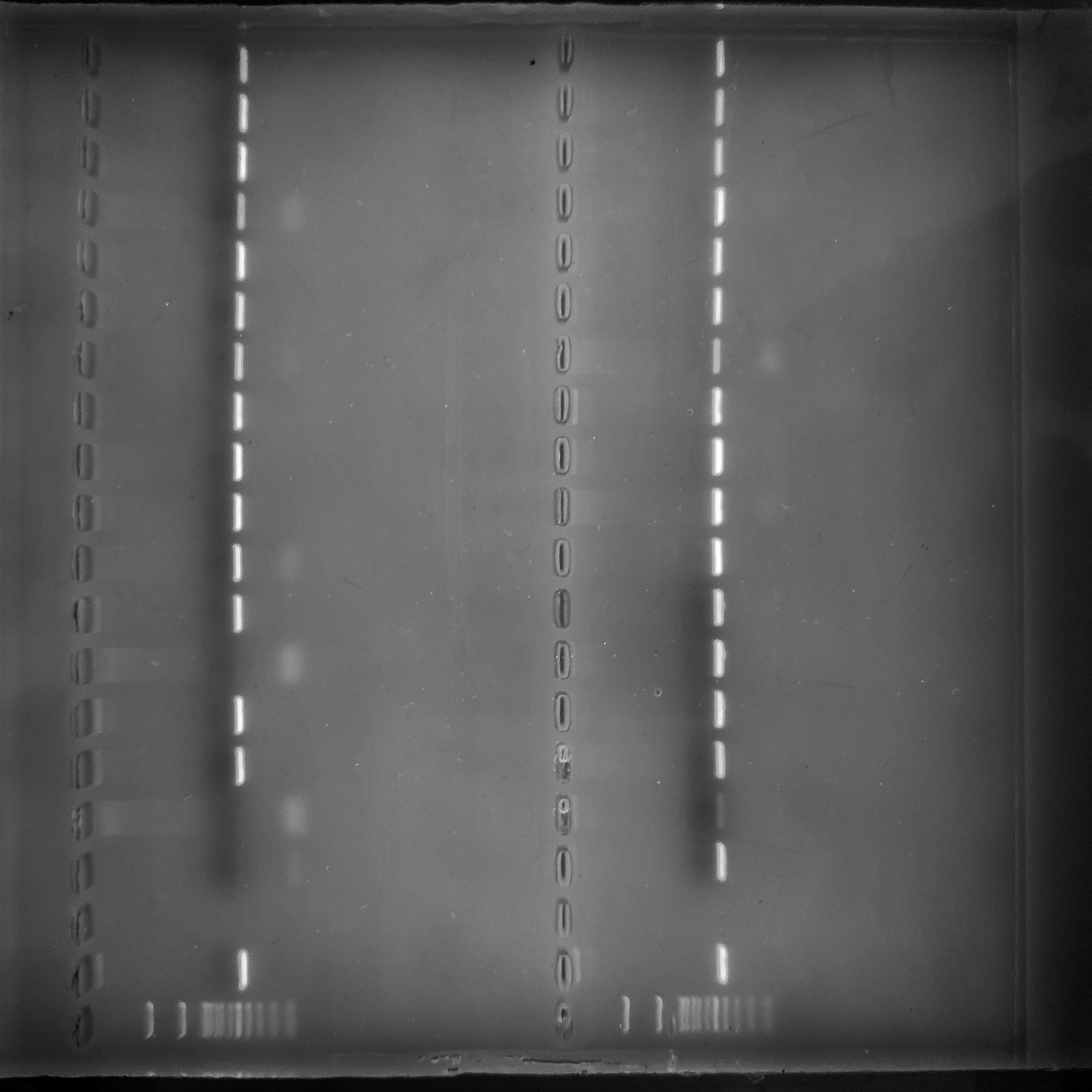

Supplement: S5 File — (JPG) [file pone.0342660.s005.jpg]

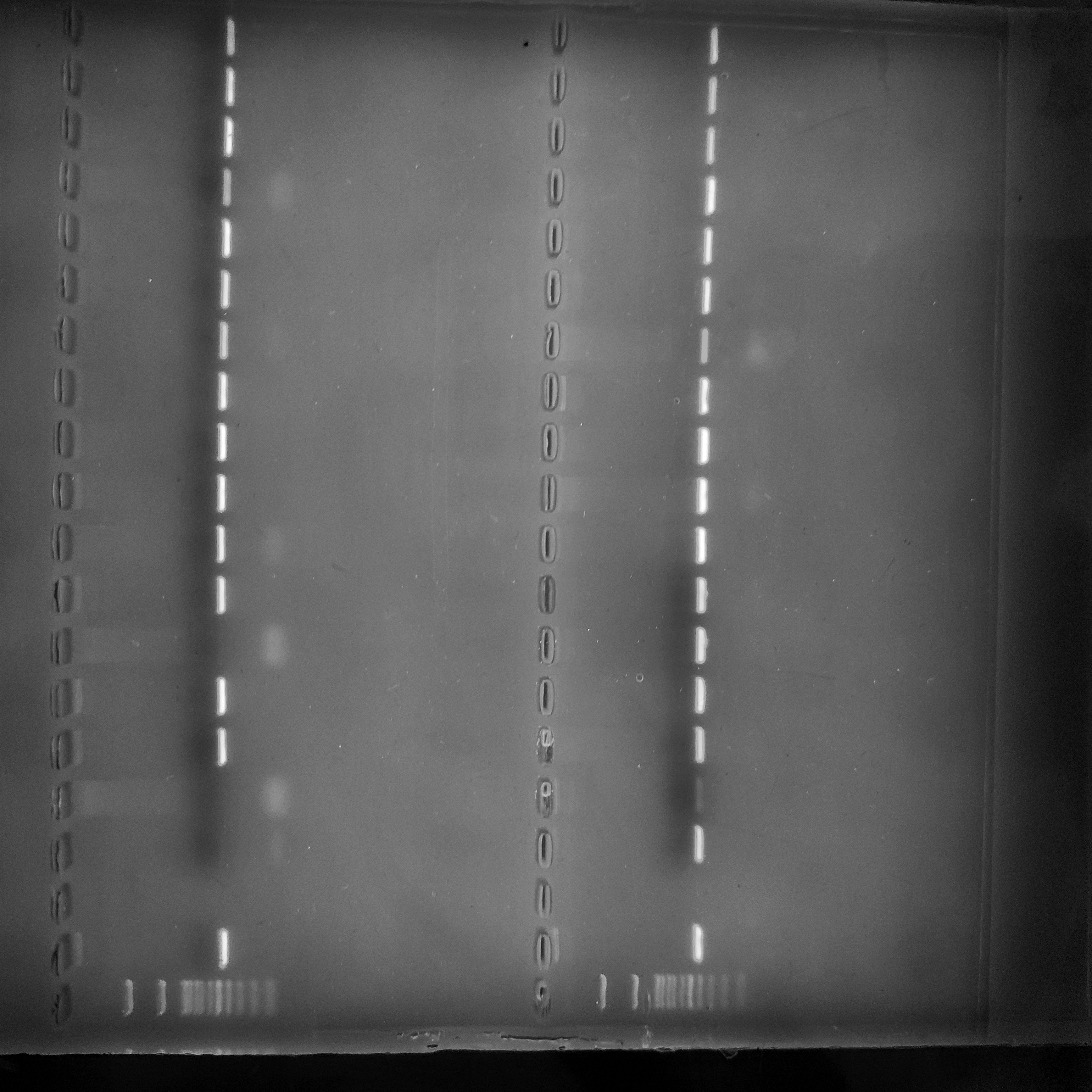

Supplement: S6 File — (JPG) [file pone.0342660.s006.jpg]

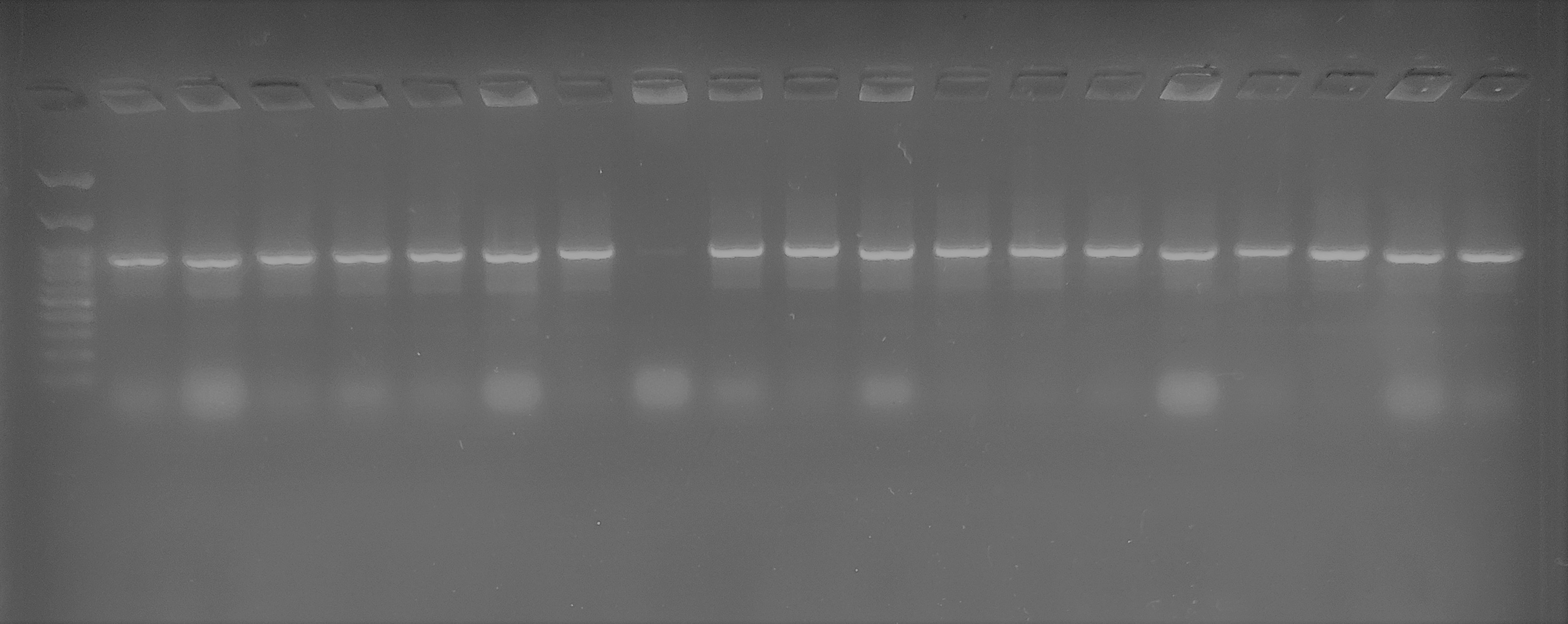

Supplement: S7 File — (JPG) [file pone.0342660.s007.jpg]

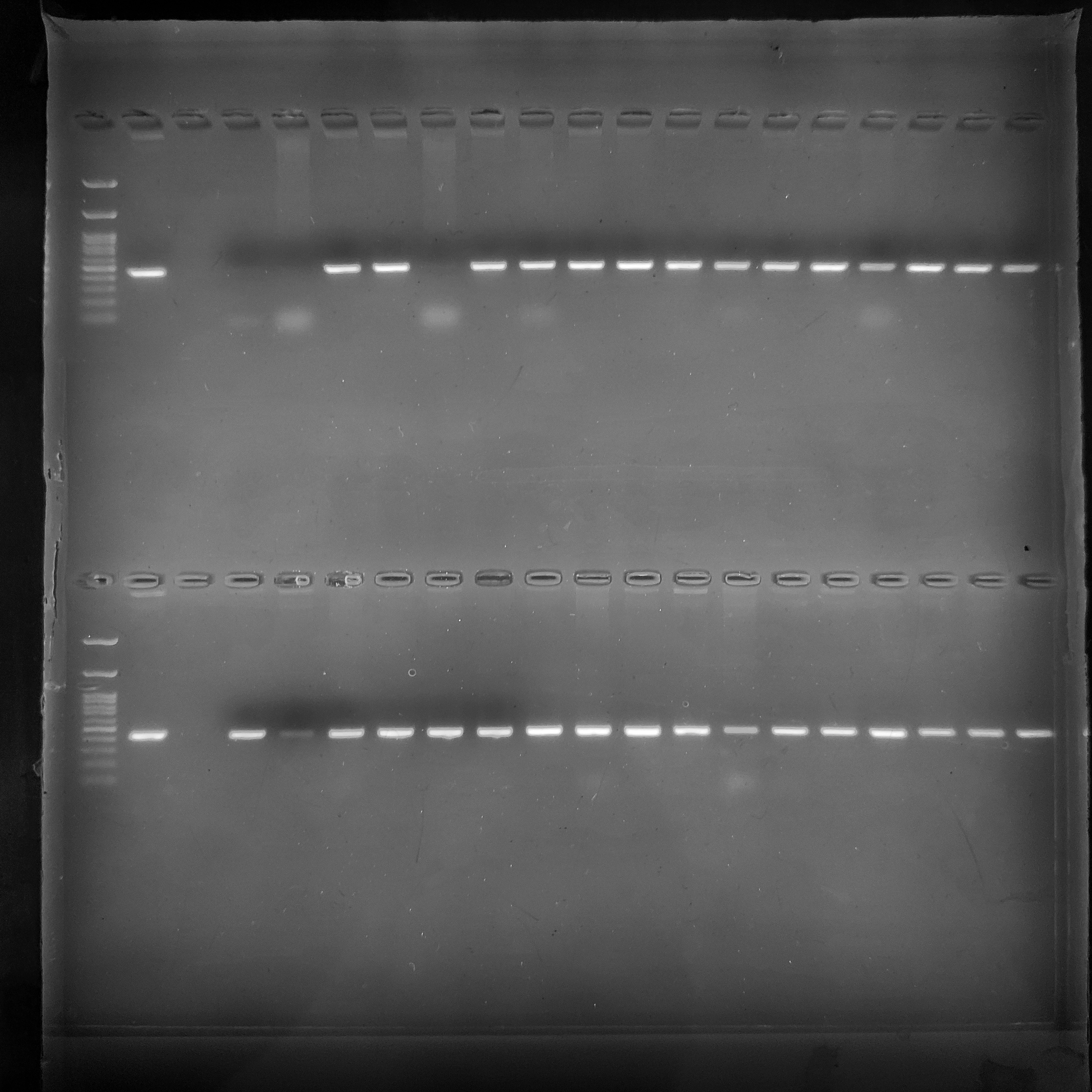

Supplement: S8 File — (JPG) [file pone.0342660.s008.jpg]
